# Outcomes after Bronchoscopic Procedures for Primary Tracheobronchial Amyloidosis: Retrospective Study of 6 Cases

**DOI:** 10.1155/2012/352719

**Published:** 2012-12-30

**Authors:** Ihsan Alloubi, Matthieu Thumerel, Hugues Bégueret, Jean-Marc Baste, Jean-François Velly, Jacques Jougon

**Affiliations:** ^1^Thoracic Surgery Department, Victor Segalen Bordeaux 2 University and Haut Lévêque Hospital, CHU de Bordeaux, 33604 Pessac Cedex, France; ^2^Pathology Department, Victor Segalen Bordeaux 2 University and Haut Lévêque Hospital, CHU de Bordeaux, 33604 Pessac Cedex, France

## Abstract

Respiratory amyloidosis is a rare disease which refers to localized aberrant extracellular protein deposits within the airways. Tracheobronchial amyloidosis (TBA) refers to the deposition of localized amyloid deposits within the upper airways. Treatments have historically focused on bronchoscopic techniques including debridement, laser ablation, balloon dilation, and stent placement. We present the outcomes after rigid bronchoscopy to remove the amyloid protein causing the airway obstruction in 6 cases of tracheobronchial amyloidosis. This is the first report of primary diffuse tracheobronchial amyloidosis in our department; clinical features, in addition to therapy in the treatment of TBA, are reviewed. This paper shows that, in patients with TBA causing airway obstruction, excellent results can be obtained with rigid bronchoscopy and stenting of the obstructing lesion.

## 1. Introduction

Primary isolated tracheobronchial amyloidosis (PTBA) is a very uncommon disease caused by aberrant extracellular deposition of amyloid fibrils, which is an inert, eosinophilic, and proteinaceous material [1]. The precise mechanism that causes amyloidosis is unknown. The natural history of this disorder and the efficacy of potential therapies have not been clearly defined. Hence we sought to investigate the long-term outcome of patients with PTBA who underwent invasive bronchoscopic treatment and stenting of the obstructing lesion.

## 2. Material and Methods

Our institute serves as a regional referral center for interventional bronchoscopy procedures. We retrospectively analyzed the medical records of all patients who were refereed to our institute for evaluation and management of symptomatic PTBA between January 2000 and October 2011. Patient data were retrieved from our local database (Epithor logiciel French cardiothoracic database society). Institutional review board approval was obtained. Informed consent for each bronchoscopy was obtained prior to the procedure. 

Each patient underwent a standard preoperative assessment, including physical examination, routine laboratory tests, spirometry, the six-minute walk test, chest radiography, and computed tomography of the chest. This paper included immunoelectrophoretic analysis of plasma and urine, salivary gland and rectal biopsies and X-rays of flat bones to look for myeloma.

An initial diagnostic flexible bronchoscopy was performed for each patient to identify the type, location, and severity of the disease. The diagnostic gold standard of amyloid is by histological confirmation through Congo red staining, which produces red-green birefringence under crossed polarised light [2]. Most tissue specimens, ranging from needle biopsies to open surgical resections, can be studied.

Rigid bronchoscopy was performed in all patients. Anesthetic induction permitted continued spontaneous ventilation by the patient until rigid bronchoscopy secured a stable airway. Rigid-bronchoscopic debulking, with adjunctive laser therapy or electrocautery, was performed for airway recanalization. If endobronchial obstruction is accompanied by marked extrinsic compression or severe stenosis, the placement of a stent may be indispensable.

Early outcomes were assessed by patient symptoms and signs, and late outcomes were assessed by patient follow-up visits, follow-up bronchoscopy, or discussion with referring physician. Patients were considered cured when free of symptoms for at least one year after the last interventional procedure. Statistical analysis descriptive data are presented as mean (±SD) or median (range). 

## 3. Results

Between 2000 and November 2011, 2758 patients were seen in our department for trachea-bronchial endoscopy. In this cohort we identified six patients with histological evidence of amyloidosis. Sex ratio was 1/1, mean age 72 years, ranging from 56 years to 83 years. All patients presented with signs and symptoms of upper airway obstruction including shortness of breath, stridor, cough, dyspnea, and wheezing and presented with typical flow volume curve that demonstrates fixed airway obstruction. 

In all patients, serum protein electrophoresis result was normal. A rectal biopsy specimen did not show any evidence of rectal amyloidosis. The diagnosis of bronchial amyloidosis was made and several investigations were undertaken. The results of urine analysis (for 24 h proteinuria and creatinuria) and protein profiles were within normal limits. An electrocardiogram, echocardiography, and abdominal ultrasound showed no abnormalities.

The plasma cell clones that underlie systemic amyloidosis are often subtle and may not be detected by bone marrow examination or immunofixation of serum and urine.

**Figure 1:**
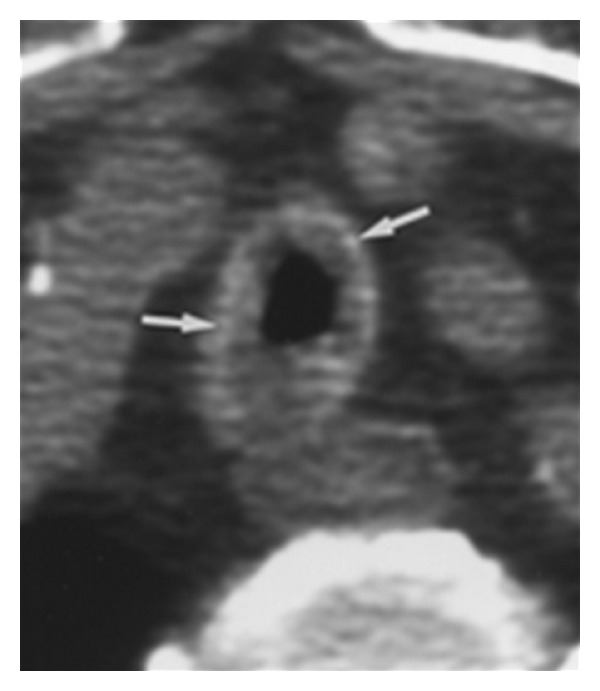
CT scan with amyloid deposits narrowing the tracheal diameter.

Flexible fibroscopy was performed in all cases; localized tracheobronchial amyloidosis was manifested by completely irregular surface of the tracheal and bronchial mucosa with prominent reddish and white-yellowish plaques, extending along the entire wall of the trachea and bronchus reducing its diameter by more than 50% with luminal narrowing and stenosis (Figure 1).

**Figure 2:**
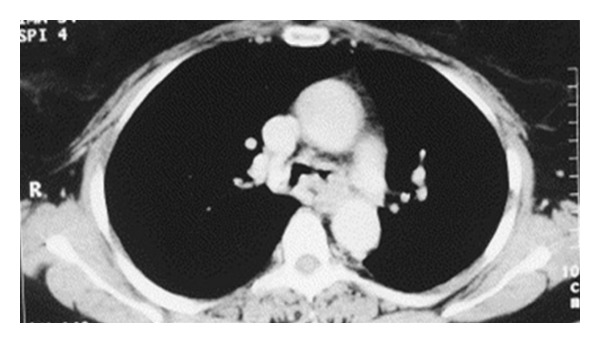
Wall thickening of the main bronchi with bilaterally luminal narrowing.

All patients underwent rigid bronchoscopy to remove the amyloid protein causing the airway obstruction. One patient presented both tracheobronchial lesions manifesting as bilateral bronchial thickening and narrowing the proximal bronchi diameter (Figure 2).

**Figure 3:**
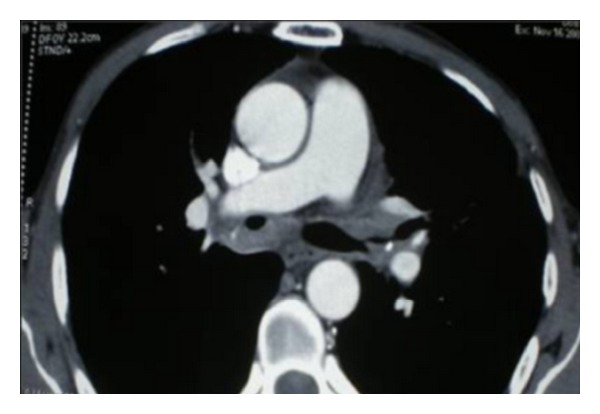
Computed tomography of the chest showing significant tracheobronchial wall thickening with narrowing of intermediate trunk.

In another patient, the main bronchi and their major divisions were the only ones affected, but in these the deposits were massive and caused extreme narrowing (Figure 3). Thus the stem bronchi to upper lobes were affected.

**Figure 4:**
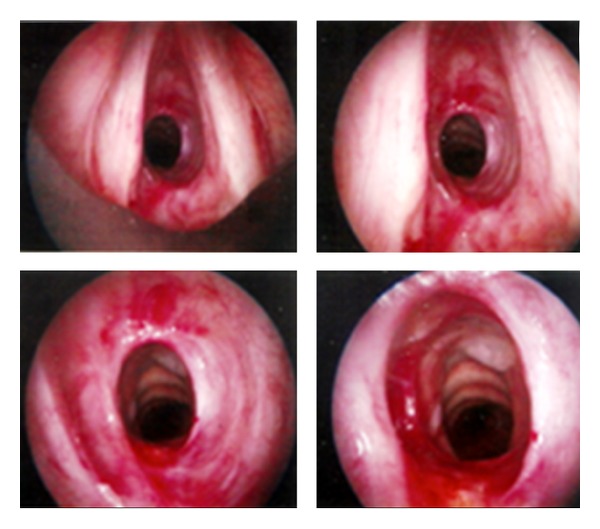
Tracheobronchial amyloidosis in a 55-year-old man. Bronchoscopic image shows subglottic stenosis and irregular mucosal thickening with diffuse nodular deposits that involve all portions of the trachea.

**Figure 5:**
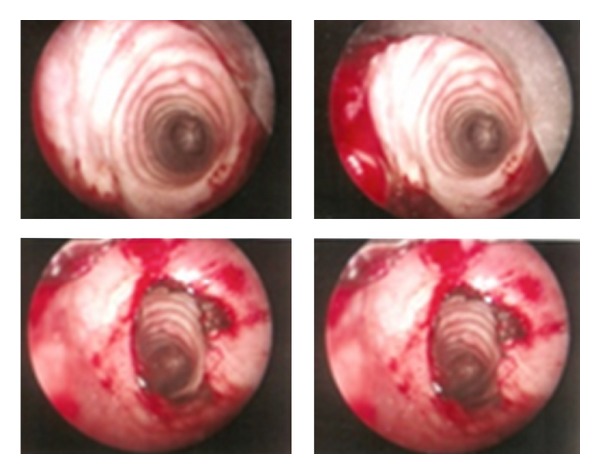
On bronchoscopy, this is the appearance of debulking with laser therapy; the left tracheal wall is edematous and narrowed.

We practiced laser therapy by neodymium:yttrium aluminium-garnet (Nd:YAG) prior to mechanical debulking, and we think that this procedure decreases intra-bronchial bleeding secondary to interventional bronchoscopy (Figure 4). The median duration of the procedure was 45 minutes (range 35–60 minutes). A satisfactory tracheal size and resolution of symptoms were obtained after multiple sessions of rigid endoscopy (three to five) under general anesthesia in four patients (Figure 5). In two others patients, the examination showed an extreme stenosis of the height trachea by tumor-like, vulnerable tissue without improvement after repeating bronchoscopy (resection and dilatation) and restenosis. A Dumon silicone stent was then inserted to alleviate the obstruction; the patients recovered well and were discharged without dyspnea. After this therapy there was an excellent clinical response; there was no stridor or rhonchi with symptomatic improvement. Follow-up bronchoscopy after six months revealed almost permeable caliber of tracheobronchial tree.

There were no intraoperative and perioperative deaths. Procedure complications were relatively manageable and included severe bleeding in three cases; laser treatment stopped the bleeding and resulted in a successful recanalization of the airways after forceps debulking. Granulation tissue formation with stenosis required recurrent laser treatments in two cases. Over a median followup of 15 months (range 6–24 months), 02 patients (33%) died. Timing from the first intervention to death ranged from 15 days to 12 months. The causes of death were respiratory failure after recurrent obstruction in one patient, and cardiac attack in other patients; death cases were due to exacerbation of the underlying condition and not directly related to the interventional procedure. Three patients were currently being followed up without any progression of symptoms. We have unfortunately no information about the last patient. The characteristics of our patients are summarized in Table 1.

## 4. Discussion

TBA is characterized by amyloid deposits primarily in the trachea and large bronchi, with extension at times into segmental bronchi. Submucosal vessels are frequently involved [1]. 

Patients with severe central airway obstruction from TBA involvement have disabling symptoms of dyspnea, respiratory distress, and obstructive pneumonia. For many of these patients, in the absence of intervention, their airway pathology may be the direct cause of death from suffocation [2]. 

Bronchoscopy is the cornerstone in the diagnosis of TBA that allows better visualization of the lesions and has the advantage of allowing excision of amyloid deposits for histopathological analysis. Diagnosis requires histological confirmation through biopsy, in which sections stained with Congo red reveal greenish birefringence under polarized-light microscopy [1, 2]. 

This paper shows that in patients with TBA causing airway obstruction, excellent results can be obtained with rigid bronchoscopy, using subjective patient symptoms to assess success in this series; 100% of patients had an immediate and significant improvement in their respiratory symptoms after bronchoscopic debridement with forceps debulking. 

All patients were treated by endoscopic debulking with two stent placements during rigid bronchoscopy, each of them with excellent clinical and functional results. In one of these patients regular endoscopic and clinical control exams were performed in the 5 years following the initial treatment, showing stable disease, requiring no further therapeutic intervention until today.

The advantages of rigid bronchoscopy in treating TBA are well known and include airway safety, the ability to perform mechanical debulking, and the ease of blood and airway secretion cleaning [3]. Laser ablation therapy was initially described as efficient in the control of local tracheobronchial amyloidosis [4, 5]. We have used Nd:YAG (neodymium:yttrium aluminum-garnet) laser therapy in all patients prior to forceps debulking with rigid bronchoscopy. We think that repeated bronchoscopic intervention with debridement of luminal obstruction with Nd:YAG laser therapy remains the standard therapeutic approach to upper and mid-airway TBA. It is thought to be preferable and safer and to reduce the major bleeding. The bleeding during bronchoscopy was described and prevalence is most likely due to fragility of submucosal vessels infiltrate with amyloid protein [6].

Overall there have been very few reports of long-term observation of TBA; unfortunately, there is no known effective treatment. Treatment options range from observation and clinical-radiological followup to forceps debulking, local aggressive radiotherapy, and laser ablation therapy. A Dumon silicone stent can be an alternative to alleviate the obstruction; many patients were treated successfully with rigid bronchoscopy and stenting and have a prompt improvement in their symptoms, and relief of impending suffocation [5, 7]. The lack of an untreated control group, or systematic followup, prevents firm conclusions about the intermediate and long-term efficacy of airway debulking. The immediately dramatic improvement in patient symptoms, seen in 100% of patients in this series, creates enthusiasm and optimism in the interventional bronchoscopists. Although, it is seems that repeated rigid bronchoscopic debridement and laser treatments did not prevent progressive airways narrowing in patients dying from TBA, in the study by Hui and colleagues [8], follow-up data were available for 7 of 14 patients with tracheobronchial amyloidosis, and 3 of these patients died of respiratory failure or recurrent pneumonia secondary to bronchial obstruction. In our series, follow-up data were available for three patients in which the disease may remain stable for five years.

## 5. Conclusion

TBA is a rare disease resulting from abnormal protein deposition within the airway submucosa. There is no cure, although forceps debulking with rigid bronchoscopy with laser therapy can improve the outlook for these patients. Despite the size of this series, in our knowledge, Bronchoscopic treatment modalities can provide durable successful results in patients with TBA with a relatively low rate of complications that can rescue the patient from imminent death and assure an improved quality of life.

## Figures and Tables

**Table 1:** Characteristics of six patients with tracheobronchial amyloidosis.

Age (y)/sex	Common symptoms	Localization/bronchoscopic description	Treatment	Followup/outcomes
70/M	Dyspnea	Tracheal/tumor	Forceps debulking Laser resection	6 months/no followup
72/F	Hemoptysis	Tracheal/nodular	Laser resection Forceps debulking	One year/died of respiratory failure
56/F	Cough, hoarseness	Laryngeal and tracheal Tumor, nodular	Laser resection Forceps debulking with stent	Two years/stable
83/F	Dyspnea, pneumonia	Tracheal and main stem bronchus submucosal	Forceps debulking Laser resection	3 years/stable
82/M	Thoracic pain, cough	Trachea, main stem bronchus Bilateral superior lobar bronchus submucosal	Laser resection Forceps debulking	Few days/died of cardiac attack (atherosclerosis)
72/M	Hemoptysis, cough, dyspnea	Bronchus/nodular, tumor	Laser resection	Five years/stable
